# Explorating the Involvement of Plasma Sestrin2 in Obstructive Sleep Apnea

**DOI:** 10.1155/2019/2047674

**Published:** 2019-11-03

**Authors:** Rong Jiang, Qiru Wang, Huifen Zhai, Xiaohua Du, Shibo Sun, Haoyan Wang

**Affiliations:** ^1^Department of Respiratory and Critical Care Medicine, First Affiliated Hospital of Kunming Medical University, Kunming, China; ^2^Clinical Medicine, 2015 Grade, Kunming Medical University, Kunming, China; ^3^Department of Respiratory Medicine, Beijing Friendship Hospital, Capital Medical University, Beijing, China

## Abstract

Obstructive sleep apnea (OSA) can lead to serious complications such as coronary heart disease and hypertension due to oxidative stress. Sestrin2 expression is upregulated under conditions of oxidative stress. This study aimed to explore whether Sestrin2 was involved in OSA. OSA and healthy control subjects were recruited and matched with age, gender, and body mass index (BMI). Plasma Sestrin2 levels were measured and compared. A multivariate stepwise regression model was used to detect the relationship between Sestrin2 and other variable factors. The Sestrin2 levels were compared between before and after four weeks treatment by nasal continuous positive airway pressure (nCPAP) in severe OSA patients. Fifty-seven subjects were divided into two groups: control group (39.33 ± 9.40 years, *n* = 21) and OSA group (38.81 ± 7.84 years, *n* = 36). Plasma Sestrin2 levels increased in the OSA group (control group 2.06 ± 1.76 ng/mL, OSA group 4.16 ± 2.37 ng/mL; *P* = 0.001). Sestrin2 levels decreased after four-week nCPAP treatment (pre-nCPAP 5.21 ± 2.32 ng/mL, post-nCPAP 4.01 ± 1.54 ng/mL; *P* = 0.004). Sestrin2 was positively correlated with apnea/hypopnea index (AHI) oxygen desaturation index, while negatively correlated with mean oxygen saturation. Moreover, these correlations remained unchanged after adjusting for gender, age, waist-to-hip ratio, and body mass index. Multiple regression analysis showed that there was an association between Sestrin2 and AHI. Our findings suggest that Sestrin2 is involved in OSA. The increase of plasma Sestrin2 is directly related to the severity of OSA. To some extent, Sestrin2 may be useful for determining the severity of OSA and monitoring the effect of CPAP. In addition, since some complications of OSA such as coronary heart disease and diabetes are usually related with oxidative stress, the role of Sestrin2 in those OSA complications needs further study.

## 1. Introduction

Obstructive sleep apnea (OSA) is emerging as a significant public health issue. It is reported that the incidence of OSA is 9–37% in males and 4–50% in females [1]. In recent years, the prevalence of OSA is increasing with the epidemic of obesity [2]. OSA is mainly characterized by repeated apnea during sleep and intermittent hypoxia [3], leading to coronary heart disease, hypertension, type 2 diabetes, cerebrovascular accident, and stroke [4, 5]. Increasing evidences show that these complications are associated with oxidative stress caused by intermittent hypoxia [6].

Sestrin2 is an oxidative stress-inducible protein regulated by p53 [7]. However, Sestrin2 performs antioxidant function through reducing overoxidized peroxiredoxins and scavenging reactive oxygen species (ROS) [8]. Its expression is elevated by DNA damage, hypoxia, and oxidative stress [9]. As a biomarker and therapeutic target, Sestrin2 is of great significance for metabolic, cardiovascular, and neurodegenerative diseases [10]. On the one hand, Sestrin2 attenuates ischemic heart by activating AMP-dependent protein kinase (AMPK) [11] and improves cerebral ischemic injury by activation of nuclear factor-erythroid 2-related factor 2 (Nrf2) pathway-mediated angiogenesis [12]. On the other hand, Sestrin2 plays an important role in metabolic diseases through inhibiting mammalian target of rapamycin complex 1 (mTORC1) [10].

OSA patients often experience repetitive hypoxia and oxidative stress during sleep [13]. We speculated that Sestrin2 was involved in OSA. Assessing this involvement might contribute to the further research of OSA complications. However, no research on this involvement had been found until now.

## 2. Materials and Methods

### 2.1. Subjects

Fifty-seven patients, including thirty-six OSA patients and twenty-one healthy control subjects, were recruited from June 2016 to June 2018 in the Department of Respiratory Medicine and Physical Examination, First Affiliated Hospital of Kunming Medical University.

The inclusion criterion was adult patients (18–65 years old). The exclusion criteria were subjects with metabolic diseases, heart failure, lung disease, cerebrovascular disease, kidney disease, immune system disease, and previous treatment for OSA. The experiment was approved by the Ethics Committee of the First Affiliated Hospital of Kunming Medical University (NO. 2016-L-51), and the subject's informed consent was obtained.

All subjects underwent a physical examination before polysomnography (PSG) test. We calculated the body mass index (BMI) according to body weight and height (BMI = weight (kg) ÷ height^2^ (m)).

### 2.2. Polysomnography

Full-night polysomnography (PSG) was performed in all subjects (Alice5, America) to record electroencephalogram (EEG) and electrocardiogram (ECG). The thermal and pressure transducer was used to measure the oronasal airflow. Oxygen saturation was monitored by pulseoximetry placed on the finger. Chest and abdomen movements were measured by strain gauges. Apnea was defined as a respiratory amplitude >90% for more than 10 s. Hypopnea was defined as a reduction of airflow >30% associated with oxygen saturation reduction >3% or arousal. All PSG records were analyzed by two specialists. The apnea/hypopnea index (AHI) of the control group was <5/h, and AHI of the OSA group was ≥5/h.

### 2.3. Pressure Titration under PSG

The overnight pressure titration was performed using an autocontinuous positive airway pressure (auto-CPAP) machine (Philips-Respironics REMstarAuto) under PSG testing. The airflow signal was replaced by a mask pressure signal, and other monitoring signals and methods were the same as the diagnostic PSG monitoring. The humidifier was routinely used in the procedure of titration. If the PSG result showed AHI <5/h, the ninetieth percentile recorded pressure was the titration pressure.

### 2.4. nCPAP Treatment

Severe OSA patients who experienced the titration pressure successfully were included in the treatment response analysis. All these subjects were treated with a nasal continuous positive airway pressure (nCPAP) machine (Resmed, S9 Escape, Australia). There was a data card in each machine to record the application time. Patients underwent the same education before nCPAP treatment. The application time was collected from the data card after four weeks of nCPAP treatment. Patients treated for more than 4 hours per day were included in the treatment analysis.

### 2.5. Blood Test

After PSG testing and nCPAP treatment, venous blood samples were obtained after overnight fasting. The plasma was extracted by centrifugation for 20 minutes at 3000 rpm and stored in a refrigerator at −80°C for testing. The ELISA kit (YAD, China) was used for Sestrin2 testing. Other blood tests including fasting blood glucose, cholesterol, low-density lipoprotein (LDL), and high-density lipoprotein (HDL) were performed by the First Affiliated Hospital of Kunming Medical University.

### 2.6. Statistical Analysis

Values were expressed as mean ± standard deviation. The normality of the data distribution was assessed by the single-sample Kolmogorov–Smirnov test. Normally distributed data were compared by independent samples *t*-test. Paired sample *t*-test was used to assess changes before and after treatment, and the rank sum test was used for nonnormal distribution data. Correlation analysis was performed using Spearson's correlation analysis and multiple stepwise regression analysis was used to determine the relationship between Sestrin2 and the other factors. Analyses were conducted using SPSS17.0.

## 3. Results

In this study, the fifty-seven subjects were divided into two groups: OSA group (*n* = 36, 38.81 ± 7.84 years old, M/F, 29/7) and control group (*n* = 21, 39.33 ± 9.40 years old, M/F, 14/7). No significant differences emerged between the two groups in gender, age, and BMI. Characteristics of all subjects are presented in Table 1. OSA patients were divided into three subgroups according to AHI: mild OSA (5/h ≤ AHI < 15/h), moderate OSA (15/h ≤ AHI < 30/h), and severe OSA (AHI ≥ 30/h). The characteristics of the subgroups are presented in Table 2.

**Figure 1:**
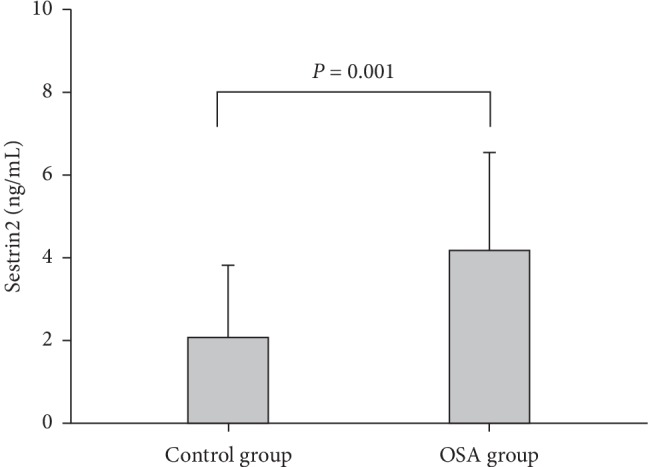
Comparison in plasma Sestrin2 level between OSA and control groups.

**Figure 2:**
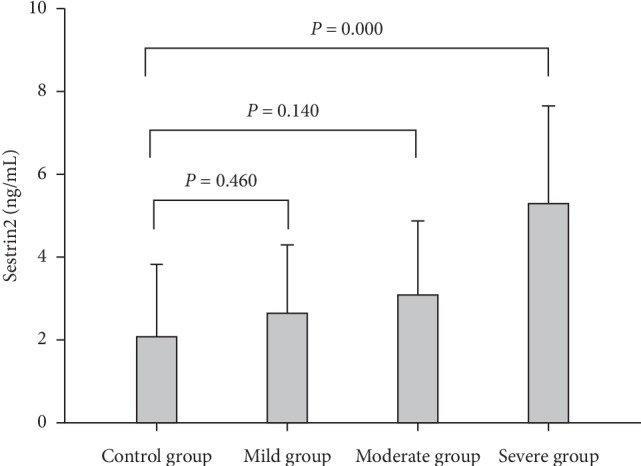
The Sestrin2 levels in mild, moderate, severe OSA, and control groups.

The Sestrin2 level was significantly higher in the OSA group than that of the control group (Figure 1). Importantly, the more severe the OSA was, the higher the Sestrin2 was (Figure 2).

Table 3 shows that Sestrin2 was positively correlated with AHI, oxygen desaturation index (ODI), HDL, and the severity of OSA, but negatively correlated with mean oxygen saturation. Moreover, these correlations existed regardless of whether gender, age, waist-to-hip ratio (WHR), and BMI were adjusted as confounding factors. Stepwise multiple regression analysis suggested that plasma Sestrin2 was associated with AHI and HDL (Table 4).

**Figure 3:**
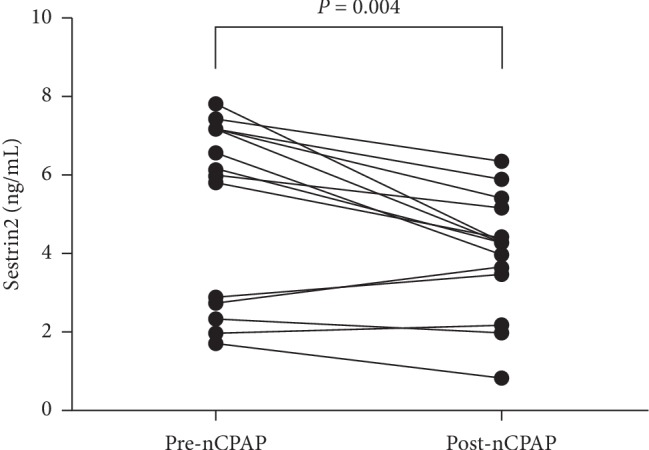
The difference in Sestrin2 level between pre-nCPAP and post-nCPAP.

Fourteen patients with severe OSA had been treated for four weeks and were included in the analysis of nCPAP treatment effect on plasma Sestrin2. Characteristics of these patients are listed in Table 5. Furthermore, plasma Sestrin2 levels decreased after nCPAP treatment (Figure 3).

## 4. Discussion

This study suggested that the level of Sestrin2 elevated in OSA patients, and there was a relationship between Sestrin2 and the severity of OSA. In addition, the Sestrin2 levels decreased after four weeks nCPAP treatment.

It was reported that intermittent hypoxia could activate and increase hypoxia inducible factor 1*α* (HIF-1*α*) by triggering nicotinamide adenine dinucleotide phosphate (NADPH) oxidase-dependent reactive oxygen species (ROS) [14, 15], and ROS is a potential inducer of the HIF-1*α* [16]. HIF-1*α* could regulate the expression of Sestrin2 [17], and the more HIF-1*α* increase, the higher Sestrin2 level is [18]. Moreover, the degree that Sestrin2 induced by HIF-1*α* is proportional to the degree of hypoxia [19]. In addition, intermittent hypoxia causes oxidative stress and activates Nrf2 [20]. This activation leads to the increase in ROS and the expression of Sestrin2 induced by transcriptional modulation of Nrf2 [21]. Due to upper airway obstruction, intermittent hypoxia occurs repeatedly in OSA during sleep [13], which causes an increase in HIF-1*α* [17, 22]. In this study, the Sestrin2 level increased in OSA and decreased after nCPAP treatment. More importantly, Sestrin2 was associated with AHI. Accordingly, we speculated that it might be intermittent hypoxia that elevated Sestrin2 levels in OSA patients. Yamamoto et al. [23] suggested that nCPAP treatment could reduce nocturnal hypoxemia and generation of ROS in patients with OSA. In addition, nCPAP treatment was effective in reducing the levels of oxidative stress [24, 25]. In the present study, the reason for the decrease in Sestrin2 level after nCPAP treatment might be that the nCPAP treatment alleviated hypoxia and oxidative stress in patients with OSA, although this speculation required further research to confirm.

Central obese increases the level of reactive oxygen or nitrogen species, causing chronic inflammation and inadequate antioxidant defenses [26]. Fat accumulation in central obesity increases NADPH oxidase activity, which leads to increased ROS production. In this study, Sestrin2 was not associated with WHR. This suggested that the increase of Sestrin2 in OSA patients might not be caused by central obesity.

Apnea events during repaid eye movement (REM) sleep are more frequent than those during non-REM sleep, which cause serious impairment of sleep structure in OSA patients [27]. REM sleep deprivation results in the increase of lipid peroxidation [28] and hippocampal oxidative stress [29]. In this study, Sestrin2 was not associated with REM, NREM, and arousal index, which meant that changes of Sestrin2 might not be related to the impairment of sleep structure in OSA.

Sestrin2 attenuates oxidative stress and inflammatory activation through an AMPK-dependent mechanism [10, 30]. Moreover, the levels of HDL increase while AMPK is activated [31]. In present study, the correlation between Sestrin2 and HDL might be associated with AMPK activation. Consistent with our view, Nourbakhsh et al. [32] also revealed that there was a positive correlation between Sestrin2 and HDL.

The main limitation of the present study was the small sample size. Another limitation was that this study was not conducted randomly. In addition, the pressure titration was performed by the auto-CPAP under PSG instead of standard CPAP titration, although it was suggested that auto-CPAP titration under PSG was also effective.

## 5. Conclusions

Sestrin2 is involved in OSA. The increase of plasma Sestrin2 is directly related to the severity of OSA. Sestrin2 level is decreased with the treatment of nCPAP. To some extent, the Sestrin2 may be useful for determining the severity of OSA and monitoring the effect of CPAP. In addition, since some complications of OSA, such as coronary heart disease and diabetes, are usually related with oxidative stress, the role of Sestrin2 in those complications of OSA needs further study.

## Figures and Tables

**Table 1:** Demographic characteristics, sleep profiles, and blood measurements of the OSA and control groups.

	Control group (*n* = 21)	OSA group (*n* = 36)	*P* value
Age (years)	39.33 ± 9.40	38.81 ± 7.84	0.821
Gender (male/female)^a^	14/7	29/7	0.195
Waist-to-hip ratio	0.87 ± 0.07	0.92 ± 0.08	0.020^*∗*^
BMI (kg/m^2^)	26.60 ± 5.31	28.28 ± 4.74	0.219
AHI (events/h)	2.47 ± 1.18	35.37 ± 20.16	<0.001^*∗*^
ODI (events/h)	1.73 ± 0.98	31.27 ± 18.57	<0.001^*∗*^
AI (events/h)	13.24 ± 7.65	36.91 ± 19.85	<0.001^*∗*^
Lowest SPO_2_ (%)	90.70 ± 1.09	79.58 ± 8.52	<0.001^*∗*^
Mean SPO_2_ (%)	93.53 ± 1.05	90.26 ± 2.92	<0.001^*∗*^
Duration of SPO_2_ < 90% (%)	0.50 ± 1.57	19.86 ± 8.76	<0.001^*∗*^
TST (min)	317.48 ± 42.53	328.44 ± 39.90	0.343
REM sleep (%)	13.52 ± 3.23	11.21 ± 3.25	0.012^*∗*^
NREM sleep (%)	86.48 ± 3.32	88.79 ± 3.25	0.012^*∗*^
Sestrin2 (ng/mL)	2.06 ± 1.76	4.16 ± 2.37	0.001^*∗*^
TG (mmol/L)	1.48 ± 0.46	1.82 ± 1.17	0.224
TC (mmol/L)	4.19 ± 0.83	4.32 ± 0.66	0.532
LDL (mmol/L)	2.30 ± 0.65	2.47 ± 0.58	0.325
HDL (mmol/L)	1.05 ± 0.33	1.00 ± 0.25	0.560
FBG (mmol/L)	4.36 ± 0.56	4.56 ± 0.58	0.213
ESS (scores)	6.76 ± 3.19	12.11 ± 4.34	<0.001^*∗*^

Values are indicated as mean ± standard deviation. OSA, obstructive sleep apnea; BMI, body mass index; AHI, apnea/hypopnea index; ODI, oxygen desaturation index; AI, arousal index; SPO_2_, pulse oxygen saturation; TST, total sleep time; REM, rapid eye movement; NREM, nonrapid eye movement; TG, triglycerides; TC, total cholesterol; LDL, low-density lipoprotein; HDL, high-density lipoprotein; FBG, fasting blood glucose; ESS, Epworth Sleepiness Score. ^a^Values are indicated as numbers. ^*∗*^*P* < 0.05, the results are statistically significant.

**Table 2:** Demographic characteristics, sleep profiles, and blood measurements of the OSA subgroups and the control group.

	Control (*n* = 21)	Mild (*n* = 7)	Moderate (*n* = 10)	Severe (*n* = 19)
Age (years)	39.33 ± 9.40	34.85 ± 8.09	39.40 ± 7.86	39.94 ± 9.40
Waist-to-hip ratio	0.87 ± 0.07	0.88 ± 0.05	0.93 ± 0.06^*∗*^	0.93 ± 0.09^*∗*^
BMI (kg/m^2^)	26.60 ± 5.31	29.62 ± 6.82	27.45 ± 2.52	28.21 ± 4.72
AHI (events/h)	2.47 ± 1.18	11.10 ± 2.66^*∗*^	22.29 ± 5.07^*∗*^	51.20 ± 13.66^*∗*^
ODI (events/h)	1.73 ± 0.98	9.32 ± 2.37^*∗*^	19.76 ± 5.46^*∗*^	45.42 ± 13.48^*∗*^
AI (events/h)	13.24 ± 7.65	16.88 ± 9.72	22.09 ± 4.17^*∗*^	52.08 ± 14.33^*∗*^
Lowest SPO_2_ (%)	90.70 ± 1.09	87.29 ± 3.30^*∗*^	83.60 ± 4.25^*∗*^	74.63 ± 8.40^*∗*^
Mean SPO_2_ (%)	93.53 ± 1.05	92.24 ± 2.01	91.67 ± 1.92^*∗*^	88.79 ± 2.91^*∗*^
Duration of SPO_2_ < 90% (%)	0.50 ± 1.57	19.77 ± 9.59^*∗*^	14.46 ± 6.15^*∗*^	22.73 ± 8.64^*∗*^
TST (min)	317.48 ± 42.53	311.43 ± 21.64	341.00 ± 46.05	328.11 ± 40.97
REM sleep (%)	13.52 ± 3.23	12.33 ± 3.58	10.30 ± 2.76^*∗*^	11.28 ± 3.39^*∗*^
NREM sleep (%)	86.48 ± 3.32	87.67 ± 3.58	89.70 ± 2.77^*∗*^	88.72 ± 3.39^*∗*^
Sestrin2 (ng/mL)	2.06 ± 1.76	2.63 ± 1.66	3.09 ± 1.77^*∗*^	5.28 ± 2.36^*∗*^
TG (mmol/L)	1.48 ± 0.46	2.09 ± 1.02	1.62 ± 0.78	1.83 ± 1.39
TC (mmol/L)	4.19 ± 0.83	4.80 ± 0.38^*∗*^	4.09 ± 0.52	4.26 ± 0.74
LDL (mmol/L)	2.30 ± 0.65	2.85 ± 0.42^*∗*^	2.31 ± 0.47	2.40 ± 0.64
HDL (mmol/L)	1.17 ± 0.34	1.00 ± 0.25	1.00 ± 0.20	1.01 ± 0.29
FBG (mmol/L)	4.36 ± 0.56	4.54 ± 0.75	4.51 ± 0.40	4.60 ± 0.62
ESS (scores)	6.76 ± 3.19	10.86 ± 2.91^*∗*^	10.80 ± 4.5^*∗*^	13.26 ± 4.51^*∗*^

Values are indicated as mean ± standard deviation. OSA, obstructive sleep apnea; BMI, body mass index; AHI, apnea/hypopnea index; ODI, oxygen desaturation index; AI, arousal index; SPO_2_, pulse oxygen saturation; TST, total sleep time; REM, rapid eye movement; NREM, nonrapid eye movement; TG, triglycerides; TC, total cholesterol; LDL, low-density lipoprotein; HDL, high-density lipoprotein; FBG, fasting blood glucose; ESS, Epworth Sleepiness Score. ^*∗*^*P* < 0.05 vs. the control.

**Table 3:** Spearman's correlations between Sestrin2 and other factors in the OSA group.

	*r*	*P* value	*r* ^a^	*P* value
Age (years)	0.080	0.642		
Waist-to hip-ratio	0.141	0.410		
BMI (kg/m^2^)	−0.120	0.486		
AHI (events/h)	0.379	0.023^*∗*^	0.401	0.023^*∗*^
ODI (events/h)	0.388	0.019^*∗*^	0.401	0.023^*∗*^
AI (events/h)	0.205	0.229	0.208	0.252
Lowest SPO_2_ (%)	−0.286	0.091	−0.314	0.075
Mean SPO_2_ (%)	−0.402	0.015^*∗*^	−0.410	0.018^*∗*^
Duration of SPO_2_ < 90% (%)	0.087	0.614	0.158	0.387
TST (min)	0.048	0.779	−0.003	0.989
REM sleep (%)	0.268	0.114	0.219	0.228
NREM sleep (%)	−0.268	0.114	−0.219	0.228
TG (mmol/L)	−0.312	0.064	−0.307	0.082
TC (mmol/L)	0.100	0.562	0.092	0.612
LDL (mmol/L)	0.207	0.227	0.223	0.213
HDL (mmol/L)	0.504	0.002^*∗*^	0.512	0.002^*∗*^
FBG (mmol/L)	−0.094	0.587	0.133	0.654
ESS (scores)	−0.022	0.889	−0.009	0.961
Severity of OSA	0.487	0.003^*∗*^	0.489	0.004^*∗*^

OSA, obstructive sleep apnea; BMI, body mass index; AHI, apnea/hypopnea index; ODI, oxygen desaturation index; AI, arousal index; SPO_2_, pulse oxygen saturation; TST, total sleep time; REM, rapid eye movement; NREM, nonrapid eye movement; TG, triglycerides; TC, total cholesterol; LDL, low-density lipoprotein; HDL, high-density lipoprotein; FBG, fasting blood glucose; ESS, Epworth Sleepiness Score. ^a^Adjusting for age, BMI, gender, and waist-to-hip ratio. ^*∗*^*P* < 0.05, the results are statistically significant.

**Table 4:** Stepwise multiple regression models of Sestrin2 levels in the control and OSA groups, adjusted *R*^2^ = 0.375.

	*B* (SE)	*B*	*P* value
Constant	−1.096 (1.022)		0.288
AHI	0.051 (0.011)	0.481	<0.001^*∗*^
HDL	3.292 (0.983)	0.356	0.001^*∗*^

OSA, obstructive sleep apnea; AHI, apnea/hypopnea index; HDL, high-density lipoprotein. Independent variables considered: age, gender, body mass index, waist-to-hip, AHI, epworth sleepiness score, arousal index, rapid eye movement sleep, triglycerides, total cholesterol, low-density lipoprotein, HDL, Fasting blood glucose. ^*∗*^*P* < 0.05, the results are statistically significant.

**Table 5:** Demographic characteristics, sleep profiles, and blood measurements of the OSA patients changeing between pre-nCPAP and post-nCPAP.

	Pre-nCPAP (*n* = 14)	Post-nCPAP (*n* = 14)	*P* value
Titration pressure (cm H_2_O)	9.71 ± 1.69	—	—
Sestrin2 (ng/mL)	5.21 ± 2.32	4.01 ± 1.54	0.004^*∗*^
Age (years)	41.7 ± 7.25	—	—
BMI (kg/m^2^)	28.74 ± 4.80	28.71 ± 4.80	0.347
Waist-to-hip	0.93 ± 0.09	0.92 ± 0.09	0.131
TG (mmol/L)	1.78 ± 1.15	1.75 ± 1.11	0.286
TC (mmol/L)	4.26 ± 0.79	4.28 ± 0.79	0.727
LDL (mmol/L)	2.55 ± 0.60	2.48 ± 0.65	0.208
HDL (mmol/L)	0.95 ± 0.18	0.97 ± 0.32	0.629
FBG (mmol/L)	4.67 ± 0.60	4.62 ± 0.52	0.272
ESS (scores)	13.50 ± 4.87	6.93 ± 1.21	<0.001^*∗*^

Values are indicated as mean ± standard deviation. OSA, obstructive sleep apnea; nCPAP, nasal continuous positive airway pressure; BMI, body mass index; TG, triglycerides; TC, total cholesterol; LDL, low-density lipoprotein; HDL, high-density lipoprotein; FBG, fasting blood glucose; ESS, Epworth Sleepiness Score. ^*∗*^*P* < 0.05, the results are statistically significant.

## Data Availability

The data used to support the findings of this study are included within the article.
